# Effects of Wire Electrical Discharge Finishing Cuts on the Surface Integrity of Additively Manufactured Ti6Al4V Alloy

**DOI:** 10.3390/ma16155476

**Published:** 2023-08-04

**Authors:** Dorota Oniszczuk-Świercz, Rafał Świercz

**Affiliations:** Institute of Manufacturing Technology, Faculty of Mechanical and Industrial Technology, Warsaw University of Technology, Narbutta 85, 02-524 Warsaw, Poland; rafal.swiercz@pw.edu.pl

**Keywords:** Ti6Al4V, finishing surface, WEDM, additive manufacturing, SLM

## Abstract

The Selective laser melting (SLM) technology of recent years allows for building complex-shaped parts with difficult-to-cut materials such as Ti6Al4V alloy. Nevertheless, the surface integrity after SLM is characterized by surface roughness and defects in the microstructure. The use of additional finishing technology, such as machining, laser polishing, or mechanical polishing, is used to achieve desired surface properties. In this study, improving SLM Ti6Al4V alloy surface integrity using wire electrical discharge machining (WEDM) is proposed. The influence of finishing WEDM cuts and the discharge energy on the surface roughness parameters Sa, Svk, Spk, and Sk and the composition of the recast layer were investigated. The proposed finishing technology allows for significant improvement of the surface roughness by up to 88% (from Sa = 6.74 µm to Sa = 0.8 µm). Furthermore, the SEM analyses of surface morphology indicate improving surface integrity properties by removing the balling effect, unmelted particles, and the presence of microcracks. EDS analysis of the recast layer indicated a significant influence of discharge energy and the polarization of the electrode on its composition and thickness. Depending on the used discharge energy and the number of finishing cuts, changes in the composition of the material in the range of 2 to 10 µm were observed.

## 1. Introduction

Titanium alloy has been increasingly used in aerospace, biomedical, or energy applications due to its properties such as low density, high heat resistance, high fatigue strength, biocompatibility, and corrosion resistance [1]. However, the machining of Ti6Al4V alloys causes many difficulties. The low thermal conductivity of titanium alloys leads to an increase in temperature in the cutting process, which causes significant wear on cutting tools [2,3,4]. The use of new technologies such as metal additive manufacturing allows for the production of geometrically complex parts or assemblies that could not be made using conventional technology. Layer-by-layer production enables the unique design and manufacturing of complex-shaped parts by lowering their weight, reducing material consumption (near zero waste technology), and reducing production costs. One of the main metal additive technologies is selective laser melting (SLM). Due to the complexity of the physical phenomena occurring in the SLM process, identifying the impact of parameters such as laser power, scan speed, scan strategy, hatch spacing, and material or powder particles on the surface integrity and the geometric accuracy of the manufacturing parts [5,6,7,8,9] is difficult. The research conducted on the optimization of the SLM process [10,11,12,13] improved the properties of the manufactured parts; however, achieving a roughness below 1 micron, which is very often required in industrial applications, is still a challenge. Despite the many advantages of using SLM in the production of parts for the aerospace or power industry [14,15], the high surface roughness, dimensional and geometric inaccuracy [16,17], defects of surface layers such as balling effect [18,19], power adhesion [20], and microcracks [21,22] in many cases require the use of additional post-processing technologies [23]. The choice of finishing techniques such as machining [24,25,26], laser polishing [27,28], mechanical polishing [29], or abrasive flow machining [30,31] is quite challenging, and it depends on the geometry of the workpiece and the required surface integrity and dimensional accuracy. Furthermore, mechanical machining or polishing Ti6Al4V alloys will face the same problems as when finishing material made in a conventional way. Therefore, new methods of finishing are sought that allow for the improvement of the properties of the surface and obtain high geometrical accuracy. One of the unconventional technologies that allows for the precise manufacturing of parts with difficult-to-cut materials is wire electrical discharge machining (WEDM). In the WEDM, electrical discharges occurring between the workpiece and the wire electrode cause local melting and evaporation of material. In the place of discharge, a crater is formed. The complexity of the physical phenomena of the material removal process causes difficulties in identifying the influence of WEDM parameters on the effects of machining [32,33,34,35,36,37,38]. A significant part of the research has focused on the modeling of WEDM and its optimization [39,40,41,42,43,44]. Nevertheless, relatively few studies have focused on the WEDM finishing of metal additive manufacturing parts. Song et al. [45] propose to use a hybrid SLM\WEDM technology to manufacture microparts. Using SLM allows for reducing the production time of manufacturing microparts, while using WEDM finishing makes it possible to obtain the required shape of microparts. Research carried out by Boban et al. [46] indicates that WEDM finishing of SLM stainless steel significantly improves surface integrity. The baling effect was removed, and the surface roughness was decreased to about 0.8 µm. As a result of the thermal effects of electric discharge, melting, and material recrystallization, grain refinement and the formation of martensite were observed, which caused an increase in the microhardness of the changed layer. Galati et al. [47] reported a reduction in surface roughness during WEDM finishing of Ti6Al4V. With decreasing discharge current and pulse time, the surface roughness decreases. The research carried out so far indicates the possibility of significantly improving the surface properties of parts using WEDM finishing technology. Although the effect of WEDM finishing cuts and discharge energy on the surface layer properties of Ti6Al4V alloy made with SLM technology has not been explored yet.

The main goal of the research is to better understand the influence of discharge energy and the polarization of electrodes in finishing passes of WEDM on the surface integrity of SLM Ti6Al4V alloy. The thermal energy produced by electrical discharges results in localized melting and evaporation of the material, which significantly modifies the surface layers. In the study, the properties of the surface integrity, including elemental composition, thickness, microstructural defects, and surface topography after WEDM fishing, were investigated.

## 2. Materials and Methods

The samples of Ti6Al4V alloy with dimensions 5 mm × 5 mm × 80 mm were made with selective laser melting (SLM) technology on the EOSINT M100 (EOS GmbH, Kreiling, Germany) machine. The specimen was made in the Z direction on the plate. The size of powder titanium particles varied in the range of 20 μm to 40 μm, with a mean value of 30 μm. Each specimen was built with the same SLM parameters and conditions at a laser power of 250 W, a scan speed of 400 mm/s, a layer thickness of 30 μm, and a hatch spacing of 100 μm. In the build chamber, oxygen was below 0.1%. In the last stage of the manufacturing process, each sample was heat-treated for one hour in a vacuum furnace with a target temperature set at 850 °C.

The finishing operation was provided on the WEDM machine Charmilles 440 (GF, Switzerland). The experimental studies were divided into two stages. In the first stage, the measuring system for analyzing voltage and current characteristics during wire electrical discharge machining has been developed. Measurement circuit characteristics are provided in previous studies [48]. Next, we conducted an analysis of the electrical discharges’ stability, which allows us to set the range of investigated WEDM parameters such as pulse time, discharge voltage, discharge current, and time interval. Furthermore, the discharge energy was calculated according to Equation (1). The list of processing parameters and conditions is presented in Table 1.

(1) $$E=\int_{0}^{t_{on}}{U_{c}\left(t\right)\cdot I}_{c}\left(t\right)dt\;(mJ),$$

where:

*U_c_*—discharge voltage (V)

*I_c_*—discharge current (A)

*t_on_*—discharge time (μs)

The negatively polarized wire electrode was used for all finishing passes except for the fourth pass, where positive electrode polarization was used. The change of polarization for the fourth pass results in an increase in the amount of eroded material from the working electrode and a simultaneous decrease in the amount of material removed from the workpiece.

**Figure 1:**
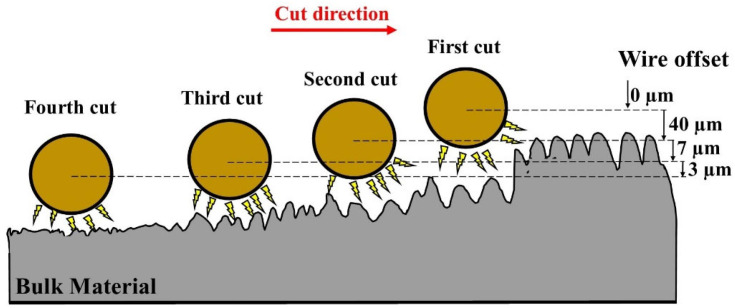
Scheme of the finishing WEDM cuts.

In the second stage, the research was focused on the analysis of the influence of discharge energy and the number of finishing cuts on the final surface layers’ properties. Samples were mounted in the clamp and cut with different offsets and discharge energies (Figure 1).

**Figure 2:**
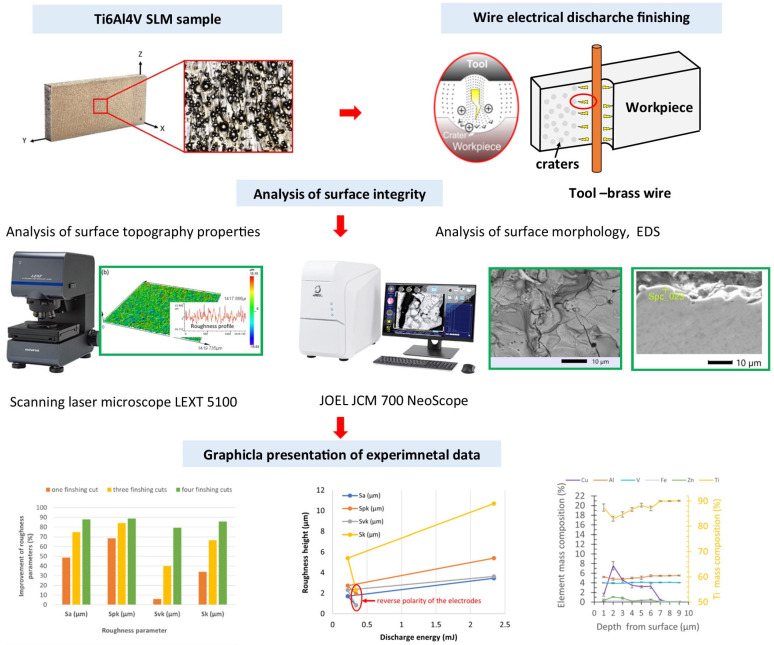
The schematic diagram of the experimental set-up.

The surface topography properties of SLM samples before and after finishing WEDM were measured with an Olympus Lext 5100 (Tokyo, Japan) confocal microscope (area 1.2 mm × 1.2 mm). The surface morphology and EDS spectrum were investigated on the JEOL JCM-7000 NeoScope (JEOL, Tokyo, Japan). The schematic diagram of the experimental set-up is presented in Figure 2.

During cutting, deionized water was supplied to the gap from the lower and upper heads of the WEDM machine. A brass wire with a diameter of 0.25 mm was used as an electrode. The experimental condition and parameters are presented in Table 2.

## 3. Results and Discussions

### 3.1. Surface Surface Morphology Properties Analyses

**Figure 3:**
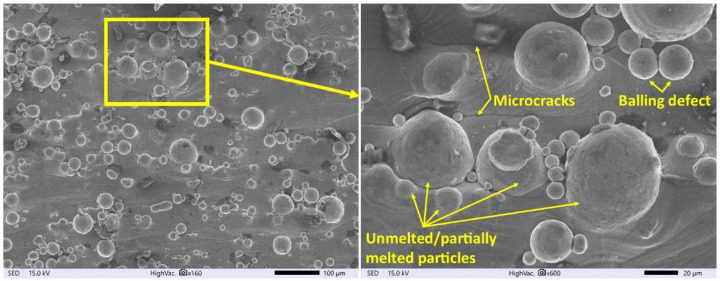
Surface topography of SLM samples of Ti6Al4V.

The surface morphology of SLM Ti6Al4V samples before and after finishing the WEDM operation was observed on the SEM Microscope. The analysis of the SEM images of the SLM surface (Figure 3) indicates numerous surface defects created during manufacturing, such as balling effects or microcracks. During SLM, the laser beam melts particles of the material and the substrate. Depending on the density of the heat source, the molten fluid dynamics change and affect the wettability of the molten material. The presence of discontinuous melted material on the surface is the result of the surface tension and viscosity of the liquid metal pool, which affect the sublimation process [49,50]. Rapid cooling of a liquid pool of metal causes shrinkage of the material, which consequently leads to the generation of residual stress. Overcoming the maximal value of material tensile strength causes the formation of microcracks on the surface (Figure 3).

**Figure 4:**
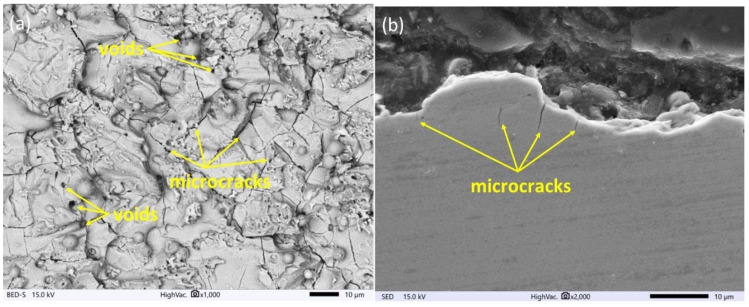
Surface morphology of AM samples after the WEDM one fishing cut for (**a**) top view of the surface, (**b**) cross section.

**Figure 5:**
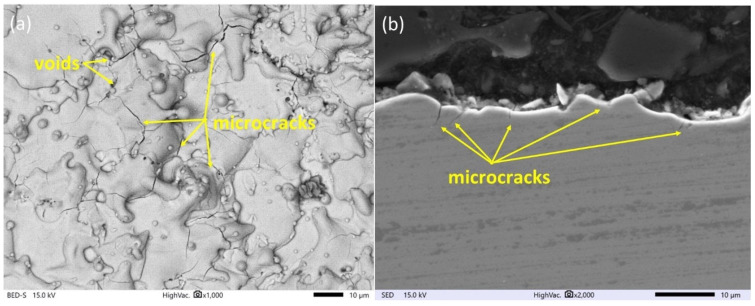
Surface morphology of AM samples after the WEDM three finishing cuts for (**a**) top view of the surface, (**b**) cross section.

**Figure 6:**
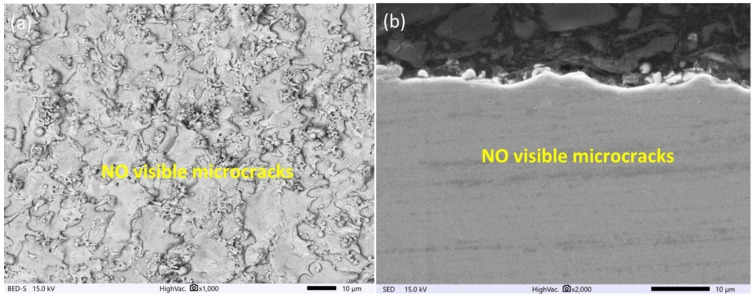
Surface morphology of AM samples after the WEDM four finishing cut for (**a**) top view of the surface, (**b**) cross section.

The surface topography has a major influence on the tribological properties of manufacturing parts and depends on the used manufacturing technology. Proposed finishing WEDM cuts for SLM samples cause significant changes in the morphology of the observed surface (Figure 4, Figure 5 and Figure 6). The unmelted powder particles and the balling defects have been removed from the surface, and a new layer with different properties has been created. Discharges occurring between the wire electrode and surface of SLM samples lead to local melting and evaporation of material. Part of the molten material that was not removed from the crater during the collapse of the discharge plasma channel and bubble gas re-solidified on the surface, and a recast layer was formed. The surface after finishing WEDM with one cut is characterized by the presence of voids and microcracks (Figure 4a). A void indicates locally rapid evaporation of the material and nonuniform resolidification of the melted material on the surface; furthermore, microcracks are visible on the entire upper surface. The analysis of the metallographic cross section (Figure 4b) shows that microcracks, in most cases, propagate perpendicularly into the material in the direction of heat dissipation. The maximum depth of microcrack propagation for one finishing WEDM cut was about 10 μm. The use of additional finishing passes (three cuts) significantly reduced the presence of micro-cracks and the depth of their propagation (Figure 5a,b). The maximum observed depth of microcrack propagation on the metallographic cross section (Figure 5b) did not exceed 5 μm. The formation of microcracks on a material’s surface is a consequence of tensile stresses. During the process of electric discharge, a thin layer of molten material that was not removed from the crater surface resolidifies into a significantly cooler core. This results in the material shrinking, an effect counteracted by the tensile stresses generated by the core material. When these stresses exceed the material’s tensile strength, microcracks occur [51]. Visible voids in Figure 4 and Figure 5 provide evidence of localized material evaporation and uneven solidification of the new material layer. These could be attributed to the existence of pores within the material layer formed during the SLM process or to the erosion of unmelted material particles with a small gap underneath them. In the case of using four finishing WEDM cuts, the microcracks have not been observed (Figure 6a,b). Reducing the energy of the electric discharge together with the use of additional trim cuts caused the recast layer to be more evenly distributed on the surface of the material, while microcracks were still observed (Figure 5a,b). The use of four finishing WEDM cuts, in which each passes the discharge energy and the amount of removed material in a single cut is reduced, allowed to obtain the surface morphology without microcracks (Figure 6a,b).

### 3.2. Surface Topography Properties Analyses

**Figure 7:**
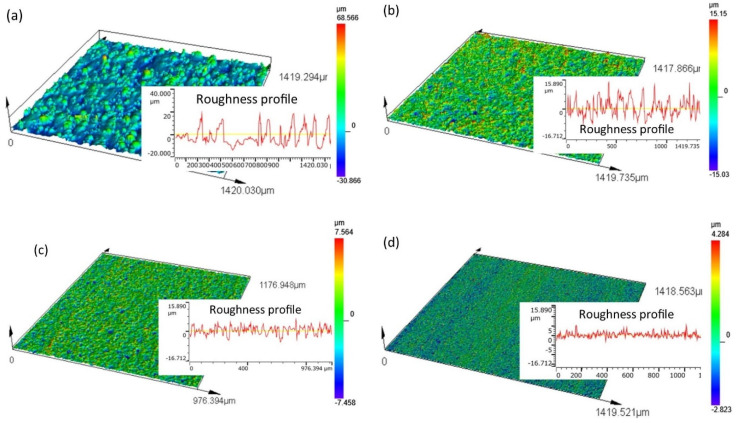
Surface topography: (**a**) SLM Ti-6Al-4V samples; (**b**) SLM samples after one fishing WEDM cut; (**c**) SLM samples after three finishing WEDM cuts; (**d**) SLM samples after four fishing WEDM cuts.

The topography of the surface after SLM is characterized by the presence of unmelted or partially unmelted powder particles (Figure 7a). The range of heights of individual peaks of the roughness is large, which leads to an uneven topography structure. The maximum observed heights of the peaks of the roughness reach 68.3 µm. The use of finishing WEDM cuts significantly affects the leveling and unification of the topography (Figure 7b–d).

In the WEDM process, for the shortest distance between the peaks of the roughness and the surface of the working electrode, the electric field intensity reaches its maximum value. In this place, at the appropriate electric voltage, the electric discharge occurs. The small amount of material is melted/evaporated, leading to a decrease in roughness. In the place of the discharge, craters are formed. Overlapping the trace of individual discharges generates the specific isotropic topography of the surface. The finishing WEDM pass reduces the height of the roughness peaks and the leveling of the surface (Figure 7b). The maximum height at which the roughness peaks after one fishing cut is below 16 µm. The use of three and four finishing WEDM trim cuts results in a further reduction of the roughness height and leveling of the surface (Figure 7c,d). The maximum observed height of the roughness peaks is 7.5 and 4.3 µm for three and four WEDM trim cuts, respectively.

The properties of the surface topography affect fatigue strength, friction processes, and abrasive wear. To characterize the properties of surfaces, the following functional parameters were chosen: the arithmetic means of the deviations from the mean, Sa (the average value of the absolute height over the entire surface), Sk (the roughness of the core), Spk (the roughness of the peak), and Svk (the roughness of the valleys). The measured surface roughness parameters are presented in Table 3.

The roughness parameters Sk, Spk, and Svk describe the Abbott–Firestone surface curve. The roughness of the valleys Svk with the lower bearing surface (SMr2) gives information about the surface’s lubrication properties. The roughness of the peak Spk can give information about the surface’s resistance to abrasion. The resistance to abrasion increases with a decrease in the roughness of the peak value. The roughness of the core (Sk) gives information about the depth of the roughness after the initial breaking-in period.

**Figure 8:**
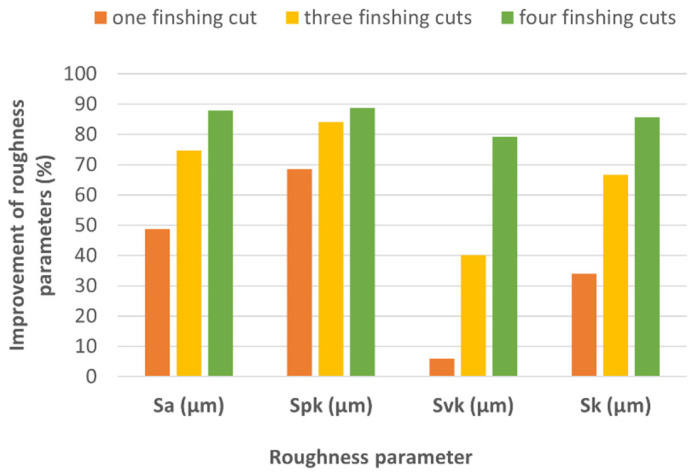
Improvement of the surface roughness parameter after finishing WEDM.

The use of a finishing WEDM trim cut in each case has a significant influence on the decrease in the roughness parameters (Figure 8). The improvement of surface roughness Sa depends on the number of trim cuts, and in analyzing cases, it has decreased by about 49%, 75%, and 88%, respectively, in one, three, and four finishing trim cuts. In the case of the roughness of the peak Spk in the first trim cut, an improvement of 68% has been achieved, and for the three and four trim cuts, 84% and 89%, respectively. A significant decrease in the value of the Spk parameter already in the first pass results from the fact that in the first cut, mainly the vertices of the roughness are removed. The material removal process for one pass also has a significant effect on the height of the roughness of the valleys Svk. A significant improvement (decrease) in the Svk parameter was observed for three and four trim WEDM cuts, and it was, respectively, 40% and 79%. Improvement of the Sk parameter was respectively about 34%, 67%, and 88% for one, three, and four trim WEDM finishing cuts. Similar results of improved roughness parameters after WEDM finishing Ti6Al4V alloy were obtained by Boban et al. [52].

**Figure 9:**
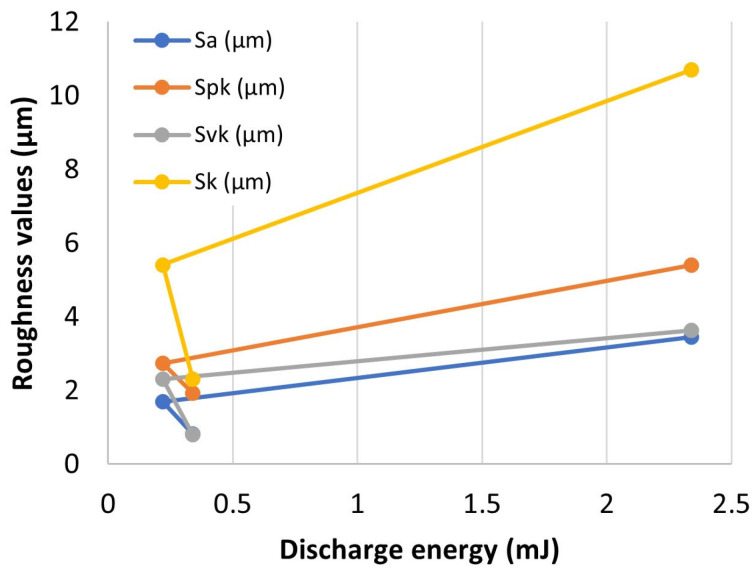
Influence of last WEDM finishing cut discharge energy on roughness height.

A significant improvement in surface roughness parameters results directly from the number of finishing WEDM cuts and the applied parameters that define the electric discharge energy, i.e., discharge pulse time, discharge voltage, and current intensity. According to Equation (1), the discharge energy was determined for an individual trim cut. The dependence of the surface roughness on the discharge energy is shown in Figure 9. An increase in the discharge energy leads to an increase in the amount of material removed in a single discharge. A larger crater is formed, which directly affects the height of the roughness. Reducing the value of the discharge energy for individual trim cuts reduces the amount of eroded material. The generated craters have a smaller height. This leads to a decrease in the value of Sa, Spk, Svk, and Sk. However, due to the construction of the generator of the wire electrical discharge machine, the reduction of the discharge energy below a certain threshold does not bring the expected effects of machining in the form of reducing the amount of eroded material in one pulse. This is due to both the limitations of the machine tool generator and the offsets used in the finishing passes. In the last pass for the planned four cuts, the offset was only 3 μm. In order to reduce the amount of eroded material in one pulse, it was decided to change the polarity of the electrodes, with the discharge energy higher than in the third trim cut (change from 0.22 mJ to 0.34 mJ). Changing the polarity of the electrodes causes more material from the working electrode (cathode) to be removed than from the workpiece (anode). With an increased discharge energy of 0.34 mJ, a reduction in the roughness parameters was obtained.

### 3.3. Surface Layer Composition Analyses

**Figure 10:**
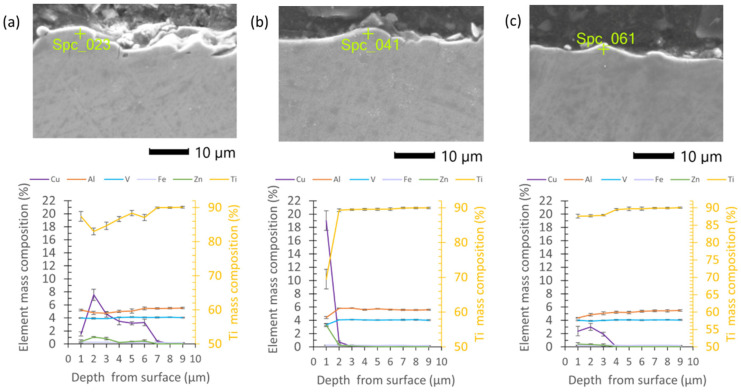
EDS element mass composition on the metallographic surface after WEDM finishing for (**a**) one cut; (**b**) three finishing cuts; (**c**) four finishing cuts.

A point EDS analysis on the metallographic surface samples after the finishing WEDM was performed. For each specimen, the point analyses were provided in the depth from the top surfaces of 1 µm to 10 µm with a step of 1 µm. The results indicate the presence of elements of the wire electrode material on the machining surface in each sample. Depending on the discharge energy and the number of passes, significant changes in the composition of the layer were observed. For the sample finishing with one cut, the elemental mass composition of the Cu is over 8% and decreases in depth to 8 µm. Furthermore, an increase in titanium from about 83% to 90% was observed (Figure 10a). The remaining elements did not undergo significant quantitative changes. The presence of zinc at a level of about 1% has been observed to be about six micrometers in depth. For a sample treated with three finishing passes, changes in composition were observed down to a depth of four micrometers, and the copper content did not exceed 4% (Figure 10b). For the samples finished with four cuts, the changes in the composition of the layer at a depth of one micrometer were the highest; the mass content of copper was about 19% and zinc about 3%, with a titanium content of 88% (Figure 10c).

**Figure 11:**
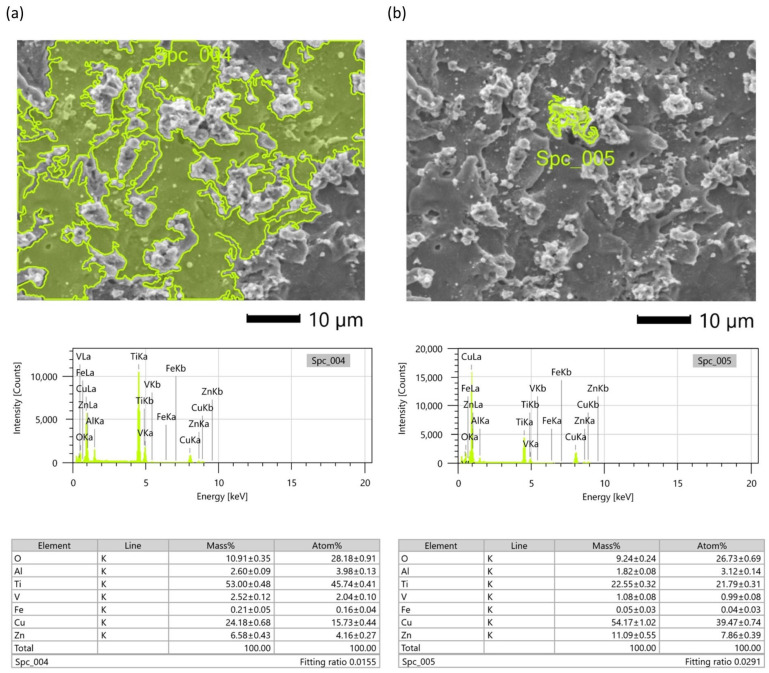
EDS spectrum on (**a**) the surface of additive manufacturing Ti-6Al-4V; (**b**) peaks of roughness, after four finishing passes.

The analysis of the composition of bottom surface morphology samples after four finishing WEDM cuts indicates (Figure 11a) a significant transfer of elements from the working electrode, including zinc and copper (over 30% of mass). The EDS analysis also indicates that the surface of the peaks of the re-solidified material (Figure 11b) contains more elements of the working electrode material than of the workpiece material (over 65% of mass). A significant increase in the content of elements in the working electrode material in relation to one and three finishing WEDM passes is directly related to the change in polarization of the working electrode in the last and fourth passes. The change in the polarity of the electrodes increases the volume of material removed from the surface of the working electrode instead of the workpiece. The intense boiling and evaporation of the electrode material during discharge cause the diffusion of material into the resolidified layers.

The presence of copper in the modified material layer after the finishing passes of electrical discharge machining is a direct result of the electric discharge process and the impact of thermal energy on both the workpiece and the working electrode. During an electric discharge, due to intense thermal processes, both the workpiece and the working electrode undergo melting and evaporation. This leads to a diffusion of the constituent particles from the brass working electrode, namely copper and zinc, to the material surface. The diffusion process is influenced by both the amount of material removed in a single pulse and the application of electrode polarization.

**Figure 12:**
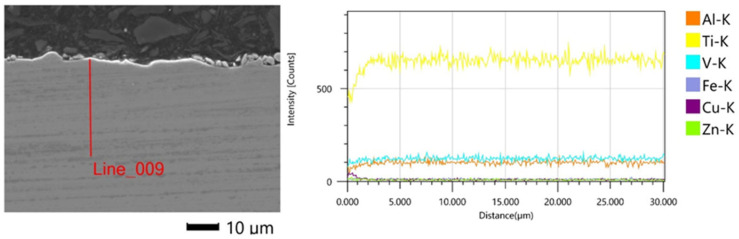
EDS spectrum on the metallographic surface of SLM Ti6Al4V alloy after four finishing passes.

EDS analysis of the metallographic samples showed the transfer of elements from the working electrode to the workpiece to a thickness of about 1–2 μm from the upper surface of the sample. Above a distance of 2 μm, no significant changes in the composition of the material were observed (Figure 12).

The results of our experimental studies suggest that to achieve the lowest surface roughness while eliminating undesired microstructural defects, it is most advantageous to use four machining passes with reverse polarity in the final pass. The use of a finishing wire cut can considerably enhance both the surface roughness and the properties of the surface layer. This research establishes a technological foundation for finishing SLM titanium alloy with the goal of achieving the desired surface roughness, surface morphology, and altered thickness of the layer composition. The achievement of a Sa roughness below one micrometer, along with the unification of the surface and the removal of defects, indicates that wire electrical discharge machining could be a promising technology for finishing SLM parts.

## 4. Conclusions

This study aimed to analyze the possibility of using WEDM on the finishing surface of SLM Ti6Al4V alloy. The influence of discharge energy and a number of finishing passes on the topography properties described by the Abbott–Firestone surface curve, roughness Sa, the composition of the recast layer, thickness, and the occurrence of microcracks were investigated. Based on the experimental results, the following conclusions were drawn:

1. The use of finishing WEDM cuts decreases the typical defects of SLM surface integrity, such as balling effects and microcracks. In the case of using four finishing passes, the occurrence of microcracks was eliminated;
2. When using four finishing passes, the smallest volume of material was removed in the last pass, which was achieved by reversing the polarity of the electrodes. This led to the least amount of thermal energy being supplied to the material, resulting in a smaller volume of material being removed and re-solidified on the surface. The reversal of polarization also reduced the temperature gradient applied to the material, which may have contributed to lower tensile residual stresses, a key cause of microcracking;
3. A significant improvement in the surface topography parameters Sa, Spk, Svk, and Sk results directly from the number of finishing WEDM cuts and discharge energy. The maximal improvement of parameters Sa, Spk, Svk, and Sk was about 88% for using four finishing WEDM cuts and the lowest discharge energy delivered to the workpiece by using the reverse polarity;
4. The thermal effects of discharges cause the change in surface morphology. The composition and thickness of the recast layer depend on the discharge energy and the number of finishing cuts. Reduced discharge energy leads to a decrease in the transfer of elements from the working electrode to the workpiece surface layer. The change in composition of surface layers was observed in the depth range of 2 to 10 µm;
5. The polarity of the electrode has a significant influence on the composition of the recast layer. The highest value of transfer zinc and copper (about 21% of mass) was observed for using reverse polarity in fourth trim cuts. However, the depth of composition change does not exceed 2 µm;
6. Further tests should be undertaken to establish the relationship between the electrical discharge energy, the volume of material removed and re-solidified, and the induced tensile stress.

## Figures and Tables

**Table 1:** Machining conditions.

SLM	Powder titanium particles	20 μm to 40 μm
	Laser power	250 W
	Scanning speed	400 mm/s
	Layer thickness	30 μm
	Hatch spacing	100 μm
WEDM	Electrode	Hard brass wire, diameter 0.25 mm
	Workpiece material	Ti6Al4V
	Discharge energy *E*	0.21–1.46 mJ
	Time break *t_off_*	3.6–18 μs
	Open voltage *U*_0_	220 V
	Dielectric	Deionized water

**Table 2:** The experimental condition and parameters.

WEDM Cutting Mode		Wire Offset (µm)	Pulse Time (µs)	Discharge Voltage (V)	Time Break (µs)	Wire Speed (m/min)
trim cut	1-trim cut	0	0.8	−80	18	8
three trim cuts	1-trim cut	0	0.8	−80	18	8
	2-trim cut	40	0.4	−80	3.6	8
	3-trim cut	7	0.2	−80	3.8	8
four trim cuts	1-trim cut	0	0.8	−80	18	8
	2-trim cut	40	0.4	−80	3.6	8
	3-trim cut	7	0.2	−80	3.8	8
	4-trim cut	3	0.2	120	3.8	8

**Table 3:** Roughness parameters after finishing WEDM.

Roughness Parameters	SLM Sample	One Finishing WEDM Cut	Three Finishing WEDM Cuts	Four Finishing WEDM Cuts
Sa (µm)	6.74	3.45	1.7	0.82
Spk (µm)	17.2	5.4	1.93	1.93
Svk (µm)	3.86	3.63	2.31	0.8
Sk (µm)	16.2	10.7	5.41	2.31
SMr1 (%)	19.4	12.5	9.09	14.9
SMr2 (%)	93	91.3	88	92.1

## Data Availability

Data will be made available on request.
